# MetaMOPE: a web service for mobile phase determination and fast chromatography peaks evaluation for metabolomics

**DOI:** 10.1093/bioadv/vbad061

**Published:** 2023-05-18

**Authors:** Dong-Ming Tsai, Ching-Yao Chang, Shih-Ming Lin, Tien-Chueh Kuo, San-Yuan Wang, Guan-Yuan Chen, Ching-Hua Kuo, Yufeng Jane Tseng

**Affiliations:** Graduate Institute of Biomedical Electronics and Bioinformatics, National Taiwan University, Taipei 106216, Taiwan; The Metabolomics Core Laboratory, Centers of Genomic and Precision Medicine, National Taiwan University, Taipei 100225, Taiwan; Graduate Institute of Biomedical Electronics and Bioinformatics, National Taiwan University, Taipei 106216, Taiwan; Department of Computer Science and Information Engineering, National Taiwan University, Taipei 106216, Taiwan; Graduate Institute of Biomedical Electronics and Bioinformatics, National Taiwan University, Taipei 106216, Taiwan; The Metabolomics Core Laboratory, Centers of Genomic and Precision Medicine, National Taiwan University, Taipei 100225, Taiwan; Master Program for Clinical Pharmacogenomics and Pharmacoproteomics, School of Pharmacy, Taipei Medical University, Taipei 110301, Taiwan; Forensic Medicine, College of Medicine, National Taiwan University, Taipei 100225, Taiwan; The Metabolomics Core Laboratory, Centers of Genomic and Precision Medicine, National Taiwan University, Taipei 100225, Taiwan; School of Pharmacy, College of Medicine, National Taiwan University, Taipei 100225, Taiwan; Graduate Institute of Biomedical Electronics and Bioinformatics, National Taiwan University, Taipei 106216, Taiwan; The Metabolomics Core Laboratory, Centers of Genomic and Precision Medicine, National Taiwan University, Taipei 100225, Taiwan; Department of Computer Science and Information Engineering, National Taiwan University, Taipei 106216, Taiwan; School of Pharmacy, College of Medicine, National Taiwan University, Taipei 100225, Taiwan

## Abstract

**Motivation:**

Liquid chromatography coupled with mass spectrometry (LC-MS) is widely used in metabolomics studies, while HILIC LC-MS is particularly suited for polar metabolites. Determining an optimized mobile phase and developing a proper liquid chromatography method tend to be laborious, time-consuming and empirical.

**Results:**

We developed a containerized web tool providing a workflow to quickly determine the optimized mobile phase by batch-evaluating chromatography peaks for metabolomics LC-MS studies. A mass chromatographic quality value, an asymmetric factor, and the local maximum intensity of the extracted ion chromatogram were calculated to determine the number of peaks and peak retention time. The optimal mobile phase can be quickly determined by selecting the mobile phase that produces the largest number of resolved peaks. Moreover, the workflow enables one to automatically process the repeats by evaluating chromatography peaks and determining the retention time of large standards. This workflow was validated with 20 chemical standards and successfully constructed a reference library of 571 metabolites for the HILIC LC-MS platform.

**Availability and implementation:**

MetaMOPE is freely available at https://metamope.cmdm.tw. Source code and installation instructions are available on GitHub: https://github.com/CMDM-Lab/MetaMOPE.

**Supplementary information:**

Supplementary data are available at *Bioinformatics Advances* online.

## 1 Introduction

Liquid chromatography–mass spectrometry (LC-MS) has become one of the primary technology used for metabolomic studies (Cubbon *et al.*, 2010; Spicer *et al.*, 2017); it is more suitable to use HILIC-based LC-MS to comprehensively identify most of the metabolites in biosamples in metabolomic studies in addition to the traditional reversed-phase LC-MS. Biofluids, such as urine, plasma, saliva and peritoneal effluent, contain polar metabolites, such as amino acids, sugars and carboxylic acids, which require an analytical platform to analyze such polar metabolites (Tang *et al.*, 2016). The typical mobile phase optimization search to establish HILIC-based LC-MS for different biofluids can be labor-intensive and time-consuming (Contrepois *et al.*, 2015). Several existing software such as TargetedMSQC and CPVA help to establish a workflow for LC-MS experiments with visualization and annotation of detected peaks (Luan *et al.*, 2020; Toghi Eshghi *et al.*, 2018).

However, existing software may not meet the need completely for the availability for metabolomics. Additionally, widely used software like XCMS and MZmine2 provide a peak table to evaluate the overall intensity of the spectrum, but may accept too many peaks without appropriate limitation on the peak quality and results in a high false positive rate (Myers Owen *et al.*, 2017). Recently, machine learning (ML) and deep learning (DL) techniques have been utilized in peak curation for LC-MS metabolomics (Gloaguen *et al.*, 2022). Metrics for peak evaluation can be used as the critical features of detected peaks for ML/DL. As a result, there is a need to develop a new software tool to determine the better mobile phase and calculate metrics for peak evaluation.

To rapidly and objectively calculate multi-metrics of peaks and determine the mobile phase for better HILIC metabolomic experiments, we developed a web tool, MetaMOPE, first to detect high-quality peaks, then identify the mobile phase conditions that produced the largest number of resolved peaks for each mobile phase with evaluating metrics regarding the morphological quality of the peaks to avoid false positives that are likely to be noises or low in abundance. With MetaMOPE, one can quickly compare the results from different mobile phases and determine the best mobile phase for the HILIC LC-MS platform. With the optimized mobile phase being determined, MetaMOPE then provides chemometrics analysis for peak evaluation for metabolomic studies. An extracted ion chromatogram (EIC) peak detection algorithm is implemented to filter out poor-quality peak features. It enables one to automatically process the repeats and determine the retention time of many chemical standards. In addition, MetaMOPE provides source codes of functions for multiple metrics written in R. Researchers can utilize functions to calculate metrics of detected peaks for further filtering, such as reducing false positive and false negative peaks. This workflow was applied to construct a reference library of 571 standard analytes for the HILIC platform.

## 2 Implementations

**Fig. 1.**
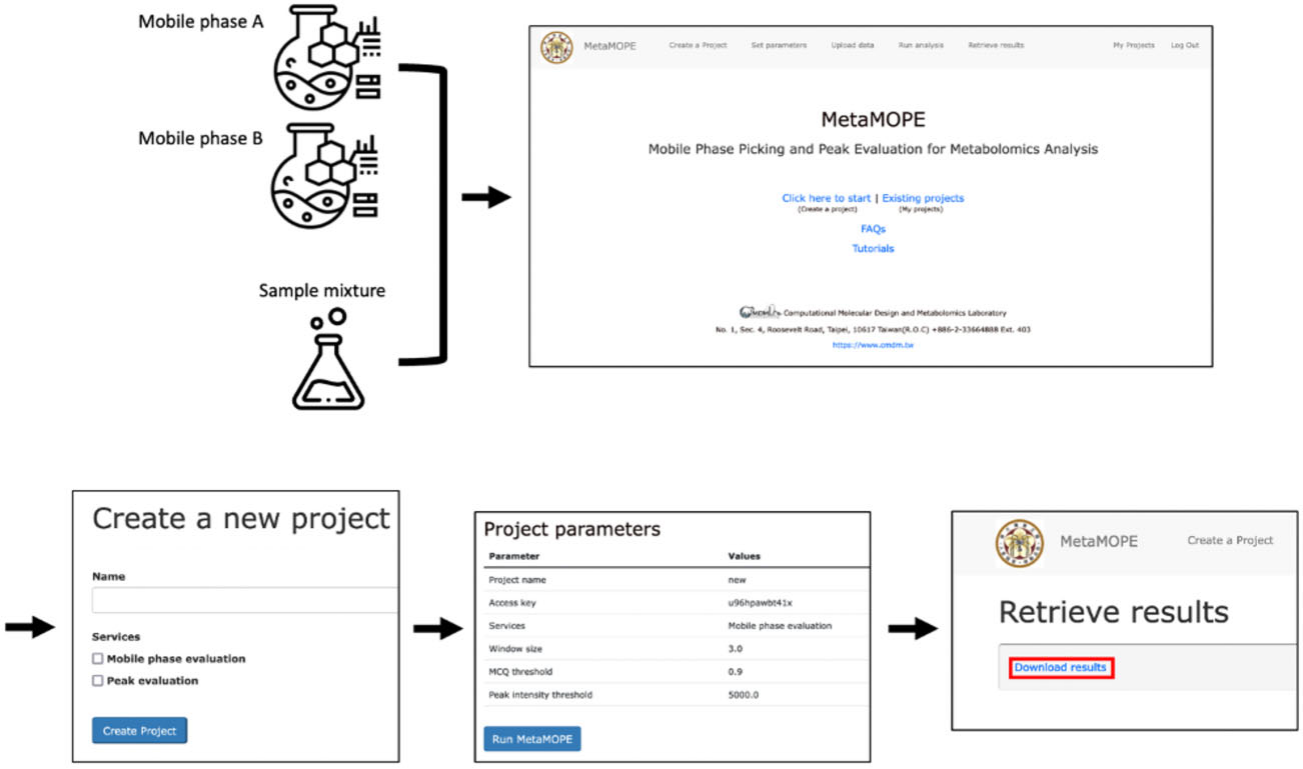
The overall working procedure and snapshots of MetaMOPE webpage

We developed MetaMOPE, a web service for determining a mobile phase of liquid chromatography and fast chromatographic peak evaluation for metabolomics. We also constructed a Docker Image for users to create their web service and deploy the application into any environment without installing the webserver. To determine the optimized mobile phase for LC-MS metabolomics analysis, we compared the quality of detected peaks in different mobile phases. We chose the one with the highest quality score as the optimized mobile phase for the sample mixture. For fast chromatographic peak evaluation, we implemented calculate metrics to assess the data quality of chromatographic peaks for metabolomics. The step-by-step tutorials can be found in Supplementary Notes. The overall workflow is illustrated in Figure 1.

### 2.1 Workflow of the mobile phase determination

**Fig. 2.**
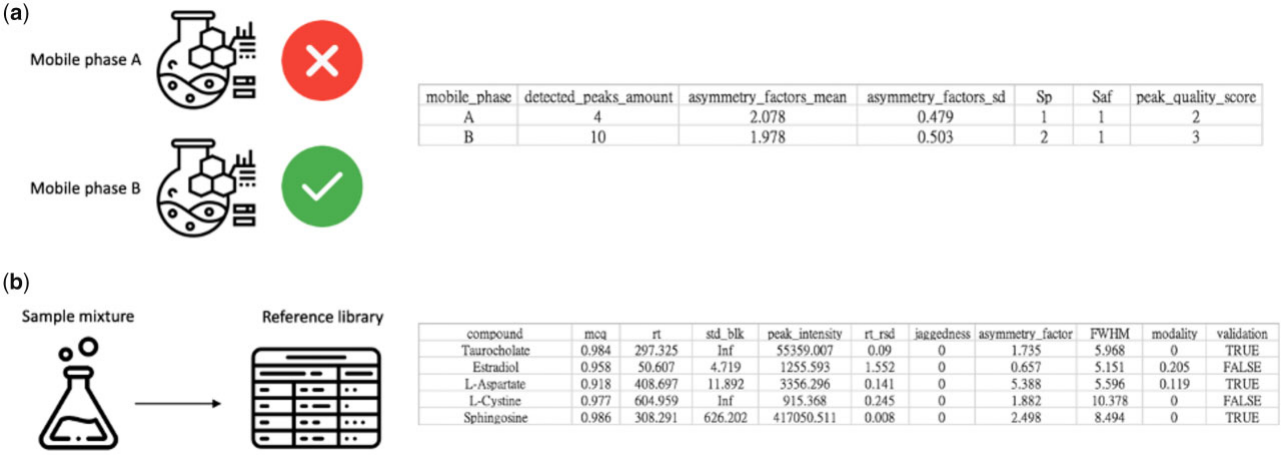
The results of (**a**) mobile phase evaluation contain a peak quality table for users to compare the separation performance between mobile phases, while the results of (**b**) peak evaluation contain a table of eight evaluation metrics and retention time of metabolites

The workflow of mobile phase determination is shown in Supplementary Figure S1a. After reading a sample’s input mzXML/mzML file, we generated standard analytes’ EIC. We calculated the maximum intensity, mass chromatographic quality index (MCQ) and asymmetry factor after IRLS (Iteratively reweighted least squares) baseline correction and Savitzky-Golay smoothing for each analyte peak. Then, we computed the number of validated peaks whose maximum intensity and MCQ are the above user-specified threshold in the experiments of each mobile phase, as well as the averaged asymmetry factor of commonly validated peaks using one-way analysis of variance. The results retrieved from the server include peak information tables regarding different mobile phases and ion modes (Supplementary Tables S1 and S2) and a peak quality score table to compare mobile phases (Fig. 2a). Two factors are considered to compare the separation performance of all mobile phases, including the number of detected peaks and the mean value of asymmetric factors of commonly separated peaks between mobile phases. The final peak quality score is the sum of the score of detected peaks (Sp) and the score of the asymmetry factor (Saf). The higher Sp indicates a larger number of peaks detected, while the higher Saf indicates better separation performance with a statistically significant difference (*P* < 0.05). The best mobile phase is the one with the highest peak quality score.

### 2.2 Workflow of the fast chromatographic peak evaluation for metabolomics

The overall workflow is illustrated in Supplementary Figure S1b. The LC/MS method is used to evaluate each standard mixture three times, and the retention time of each reference analyte is generated from the EICs of each analyte in the three replicates. The extracted ion chromatograms of each chemical standard were retrieved (Supplementary Fig. S2), followed by baseline correction and smoothing of EIC using the IRLS algorithm and Savitzky-Golay filter. Only the peaks that satisfy the following criteria are recorded: the ratio of peak intensity to blank intensity (Std/Blk) is greater than a user-specified threshold *r*, the relative standard deviation (RSD) of the retention time of the detected peaks of the three replicates is less than a user-specified threshold c, and the peak intensity is larger than a user-specified threshold *I*. MetaMOPE generated the following eight metrics for each peak: (i) MCQ, (ii) the maximum intensity, (iii) the RSD of retention time, (iv) the ratio of standard over blank (Std/Blk) intensity, and those concerning the morphology of the peak, including (v) jaggedness, (vi) asymmetry factor, (vii) FWHM and (viii) modality (Supplementary Notes). The retention time information of the standard analytes is also provided. The final results of eight evaluation metrics and the retention time of each metabolite can be retrieved from the server (Fig. 2b). The column of validation decides whether the metabolites in the sample mixture are validated by the user-specified thresholds. Users can evaluate the chromatographic peak quality and determine the retention time of validated metabolites as a reference library.

## 3 Case study

We deployed MetaMOPE to select mobile phases and build reference libraries for targeted metabolomics research to demonstrate the functionalities. To determine the optimized mobile phase for HILIC metabolomic analysis, we compared the quality of detected peaks in two mobile phases with a mixture of 20 chemical standards in the samples and chose the one with the highest quality score (see Supplementary Tables S1 and S2). After selecting the optimized mobile phase for selected chemical standards, we examined the repeats of 20 chemical standards as the example for chromatographic evaluation of construction of reference library for metabolomics (see Supplementary Table S3 for metrics).

### 3.1 Using mobile phase determination for the HILIC column platform

We ranked the mobile Phases A and B according to the sum of the score of the detected peaks (Sp) and the averaged asymmetry factor (Saf) score to determine the optimized mobile phase. In Figure 2a, mobile Phase A has four detected peaks, and mobile Phase B has 10 detected peaks. The Sp of mobile Phases A and B are one and two points, respectively. Mobile Phase B is better than mobile Phase A. The Saf of the two mobile phases is equal (both Saf are one) since there is no statistically significant difference. The best mobile phase is mobile Phase B, with the highest peak quality score (three, two points for Sp and one point for Saf).

### 3.2 Using fast chromatographic peak evaluation to construct a reference database

When the optimized mobile phase is selected, a chemometric workflow is applied to build the reference analyte library. By default, the thresholds *r*, *c*, and *I* was set to 6, 15%, and 1000, respectively. Boudah *et al.* (2014) chose ratio two to filter the peak feature for the biological sample to the blank sample, and we chose ratio 6 for Std/Blk index r to reduce false-positive detections. Other parameters (*c* and *I*) are commonly used for quality control samples and may help to pick out high-quality peak features (Godzien *et al.*, 2015). Figure 2b shows the results of five constituent metabolites in the reference analytes. The results of taurocholate, l-aspartate and sphingosine satisfy the abovementioned criteria to be validated as the reference library (see Supplementary Table S3 for detailed metrics). Estradiol dropped out due to low Std/Blk (4.719 < 6), while l-cystine dropped out due to low peak intensity (915.368 < 1000).

## Supplementary Material

vbad061_Supplementary_Data

## Data Availability

The data underlying this article are available in Figshare, at https://doi.org/10.6084/m9.figshare.22827152.
