# Supplementary material for: MetaMOPE: a web service for mobile phase determination and fast chromatography peaks evaluation for metabolomics
Source: Bioinform Adv. 2023 May 18;3(1):vbad061. doi: 10.1093/bioadv/vbad061 (PMC10206287; doi:10.1093/bioadv/vbad061)
Supplement: vbad061_Supplementary_Data [file vbad061_supplementary_data.docx]

**Supplementary Material**

MetaMOPE: a web service for mobile phase determination and fast chromatography peaks evaluation for metabolomics

Dong-Ming Tsai^1,2^, Ching-Yao Chang^1^, Shih-Ming Lin^3^, Tien-Chueh Kuo^1,2^, San-Yuan Wang^4^, Guan-Yuan Chen^5^, Ching-Hua Kuo^2,6^, Yufeng Jane Tseng^1,2,3,6*^

^1^ Graduate Institute of Biomedical Electronics and Bioinformatics, National Taiwan University, Taipei, Taiwan,

^2^ The Metabolomics Core Laboratory, Centers of Genomic and Precision Medicine, National Taiwan University, Taipei, Taiwan,

^3^ Department of Computer Science and Information Engineering, National Taiwan University, Taipei, Taiwan,

^4^ Master Program for Clinical Pharmacogenomics and Pharmacoproteomics, School of Pharmacy, Taipei Medical University,

^5^ Forensic Medicine, College of Medicine, National Taiwan University, Taipei, Taiwan,

^6^ School of Pharmacy, College of Medicine, National Taiwan University, Taipei, Taiwan.

1. **Supplementary Notes**
   1. **Working guide**

MetaMOPE contains two sequential analysis processes: mobile phase determination and fast chromatographic peak evaluation to build a reference library of validated metabolites. Examples were We used 20 chemical standards to demonstrate with MetaMOPE for mobile phase determination and chromatographic peak evaluation as the examples. The data are displayed in Table S1, S2 and S3.

Yet, these two analysis processes are implemented independently. When creating a new project, users must first choose one of the services to run. Different types of services require different parameter settings and input files. The step-by-step guides to setting the parameters and generating the input files are described below.


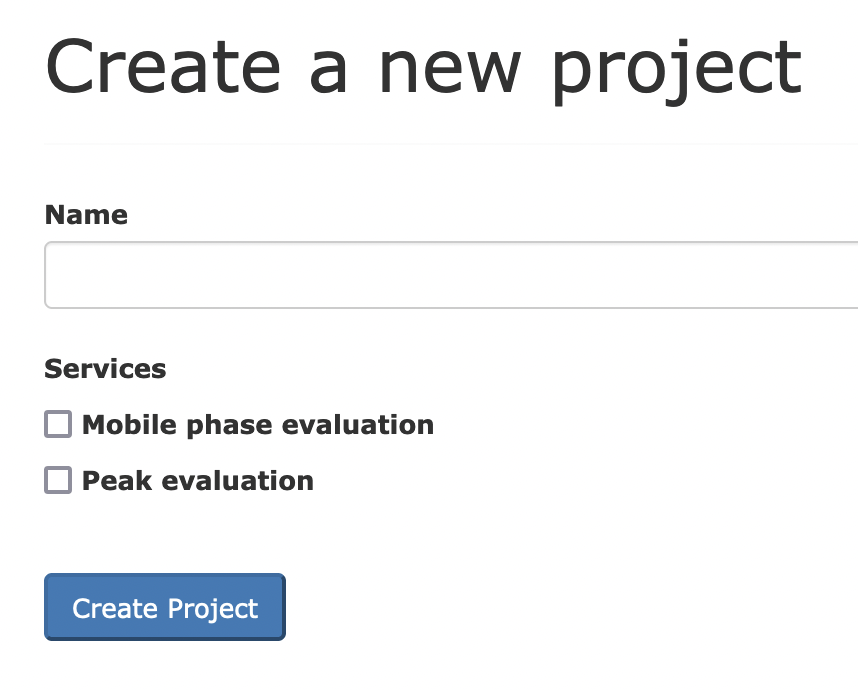


- - 1. **Mobile phase determination**

Users can assign three analysis parameters to determine an optimized mobile phase: mass chromatographic quality index (MCQ) threshold, peak intensity threshold, and window size calculate MCQ. Users can also apply the default values of 0.9, 5000.0, and 3.0, respectively. Also, the process requires a CSV file containing all the standard analytes and their mass-to-charge ratio (m/z) in both positive and negative ion modes. The two figures below are screenshots of the parameters setting page, which includes detailed descriptions of the CSV file format. Users need to follow the column format or download the CSV file with the standard format provided on the web page to record the required information to generate the results successfully.


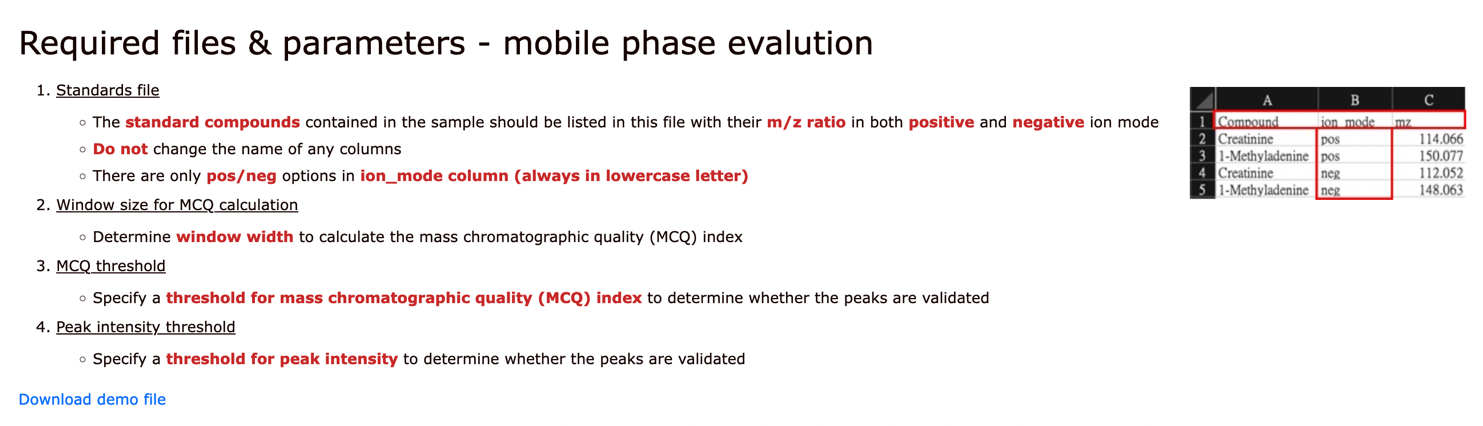


Standard format of the CSV file


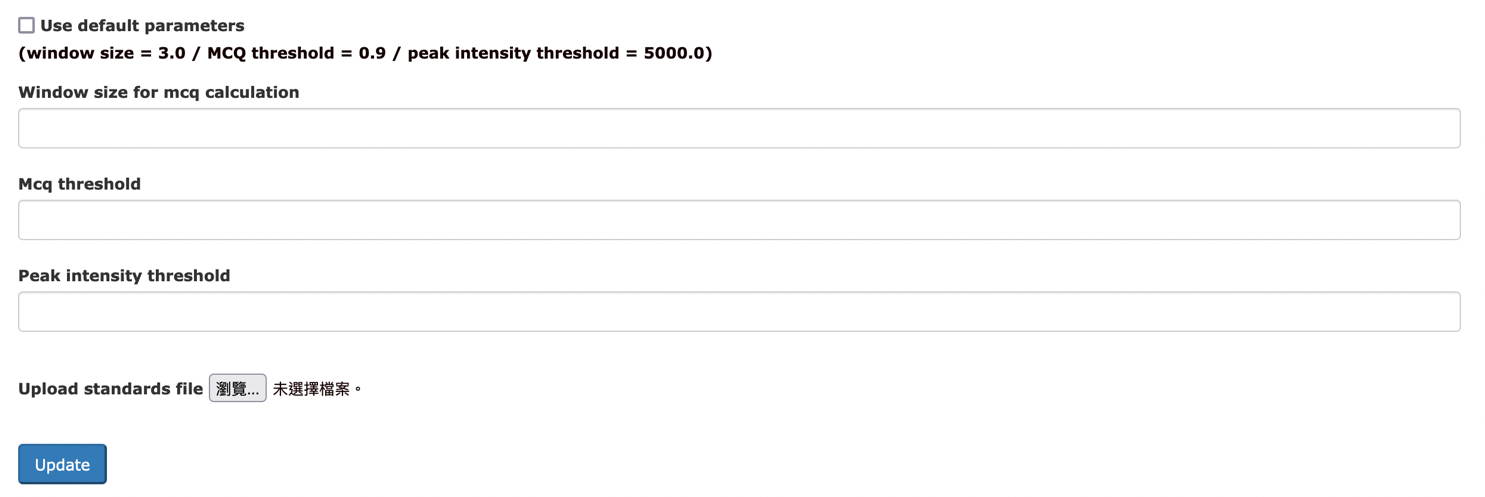


After setting the parameters, users will be guided to the data uploading page to upload the mzXML files of metabolomic experiments on the HILIC LC-MS platform using different compositions of mobile phases. Each mobile phase takes one uploading form, in which users need to assign the name of the mobile phase and its mzXML files. There are also some naming rules for the input file described on the web page. Since the size of the files to be uploaded is quite large, it may take a while to upload all the mzXML files.


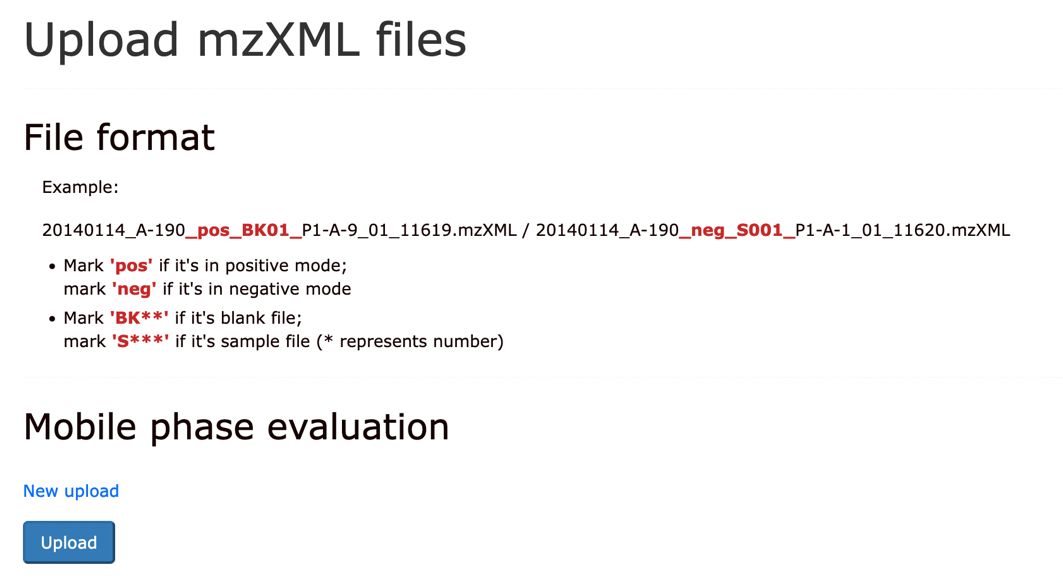


Click to add results of new mobile phases

As shown in the right figure below, users can run the analysis with all the parameters and files uploaded. The analysis process may take a few minutes to finish. Afterward, users can retrieve the results (.zip) by clicking the download link shown in the left figure below.


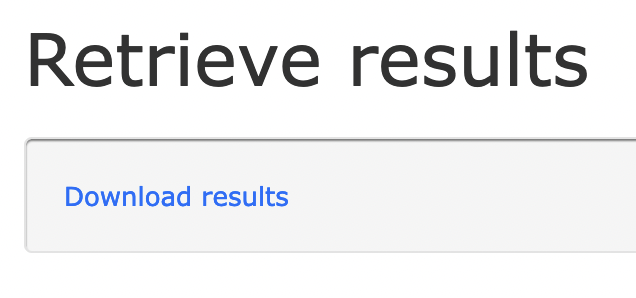


The downloaded results are zipped into a compressed file. It contains the peak information tables of all the standard analytes regarding the separation performance (MCQ, peak intensity and asymmetry factor) of each mobile phase in both positive and negative ion modes. In addition, a CSV file comparing the overall separation performance of mobile phases is attached. It compares the separation performance based on the amount of detected peak and the mean asymmetry factor, both of which are then rank into a peak quality score. The mobile phase with the highest peak quality score is the optimized one to the sample mixture.

- - 1. **Fast chromatographic peak evaluation**

All the above working procedures of mobile phase determination can be applied to the process of peak evaluation, only that there are five parameters to be set, including MCQ threshold, peak intensity threshold, window size, the threshold of the ratio of standard over blank signal, and the threshold of the relative standard deviation of retention time. In addition, this process requires one more input CSV file, which should record the names of all mzXML files, including blank and samples.

The resulting file contains a CSV file. In the CSV file, all target metabolites are listed in rows with the calculated values of 8 peak metrics (Please see section 1.2 of supplementary information). Users can overview all target metabolites with peak metrics.

- 1. **Eight metrics evaluation of chromatographic peak**
     1. **MCQ**

The mass chromatographic quality index is the similarity between the original mass chromatogram and the smoothed mean-subtracted version. It is used to assess the quality of potential peaks and eliminate the noise peaks. If the MCQ index is under the user-specified threshold, which is 0.9 by default, the peak is regarded as a noise peak. This work calculates the mass chromatographic quality index by the component detection algorithm (CODA).

- - 1. **RSD of RT**

The relative standard deviation of the retention time of the candidate peaks is used to evaluate the morphology of a candidate peak. If it is above the user-specified threshold, the width of the candidate peak may be too large to be a standard signal. By default, the threshold is set to 1.5.

- - 1. **Std/blk**

The ratio of the intensity of standard analytes over blank solution is calculated to eliminate some minor signals. The candidate peak can only be validated as a standard signal when the ratio is above the user-specified threshold. The threshold is set to 6 by default, which means the intensity of common analytes must be six times larger than that of the blank solution.

- - 1. **Jaggedness**

Jaggedness is a measurement of how often a peak changes its direction. A jaggedness closer to 0 refers to a smooth peak, while a jaggedness closer to 1 is considered a noisy peak.

- - 1. **Asymmetry factor**

The asymmetry factor refers to the symmetry of a chromatographic peak. It is defined as the horizontal distance of the measurement point on the center line to that on the back slope divided by the horizontal distance of the measurement point on the center line to that on the front slope, where all the measurement points are at the 10% of the maximum peak height. An asymmetry factor larger than 1 indicates a tailing; however, an asymmetry factor smaller than 1 indicates a fronting peak. Both tailing and fronting peaks suggest a poor separation performance.

- - 1. **FWHM**

The full width at half maximum is defined as the width at the level of half of the maximum intensity of the peak. It is used to measure the resolution in mass spectrometry, where a smaller FWHM indicates a better resolution, which results in a better separation of peaks.

- - 1. **Modality**

Modality refers to the largest dip in a peak, which indicates a poor morphological quality of a peak. It is the depth of the largest dip divided by the peak height.


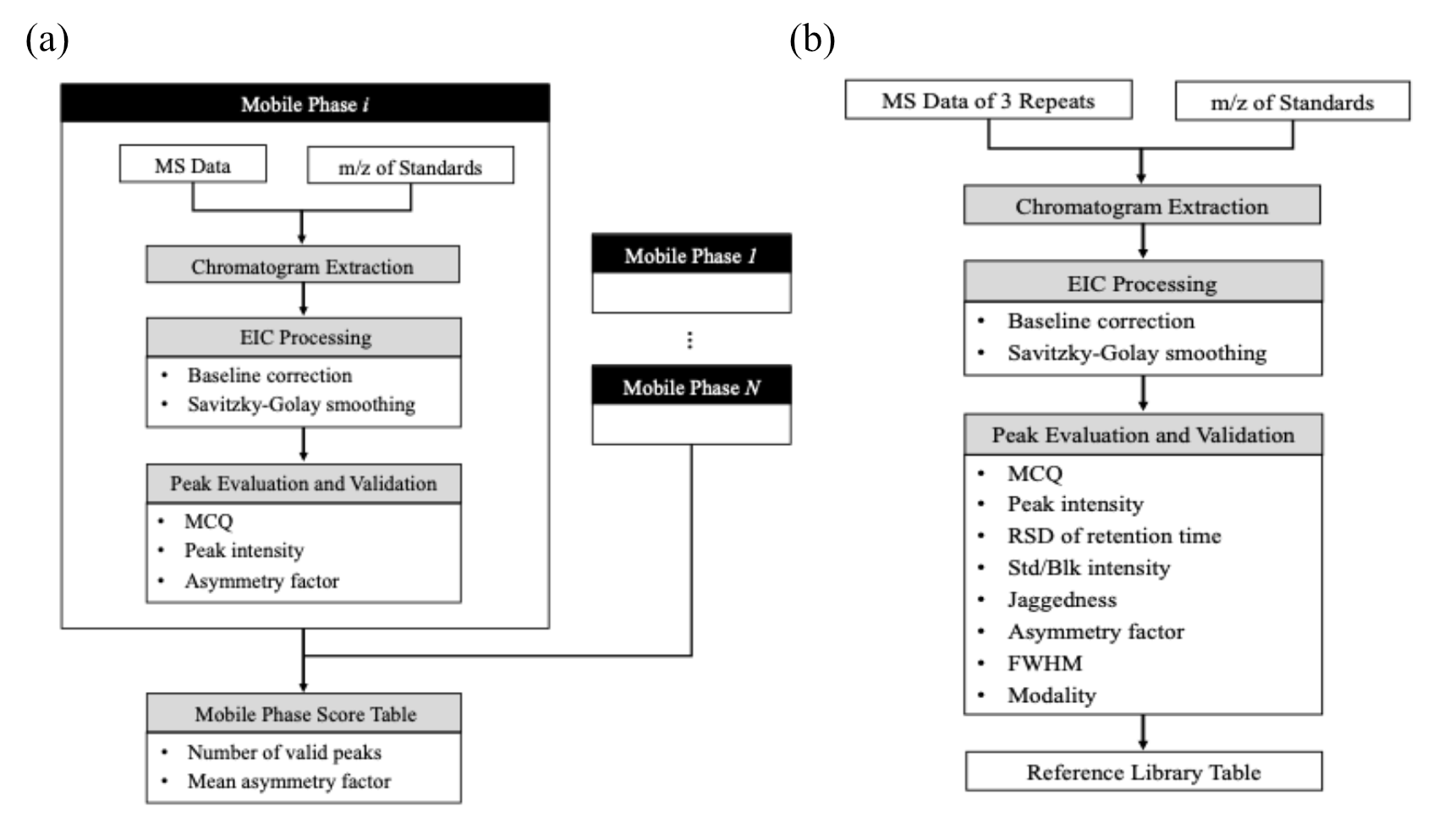


**Figure S1. a) mobile phase determination and b) fast chromatographic peak evaluation for metabolomics.**


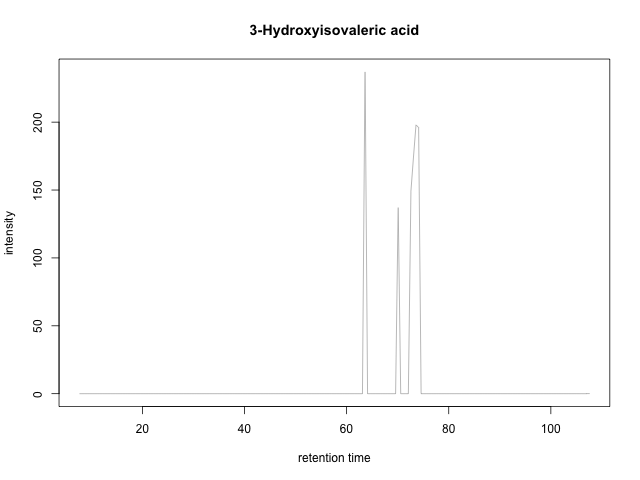

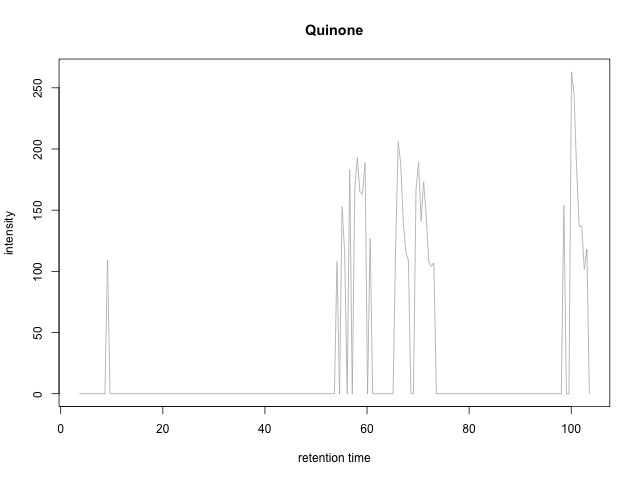

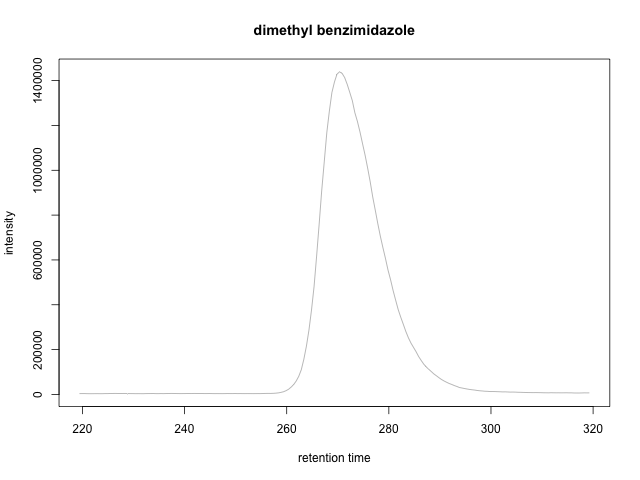

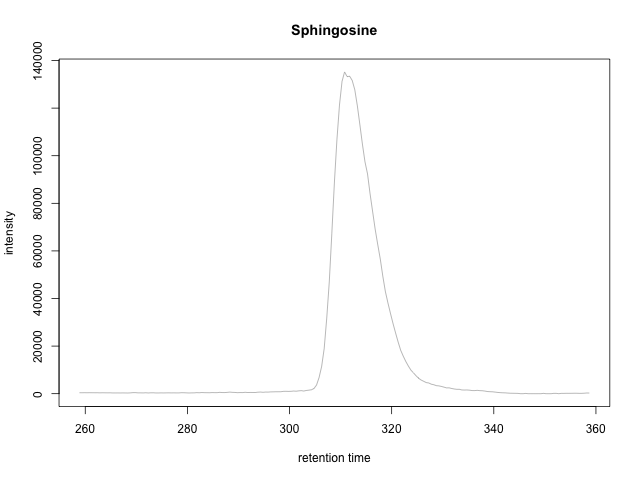

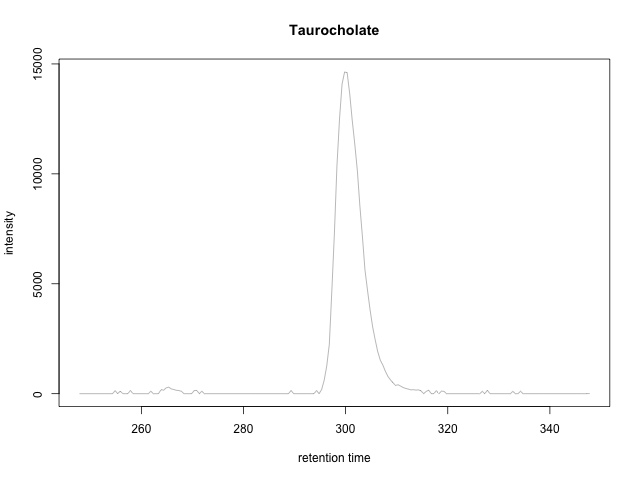


**Figure S2. Visualized Extracted ion chromatograms of some chemical standards in the sample mixture. The peak intensity of 3-hydroxyisovaleric acid and quinone are not above the previously specified threshold to be validated.**

| **Compound** | **MCQ** | **peak_intensity** | **asymmetry_factor** |  | **Compound** | **MCQ** | **peak_intensity** | **asymmetry_factor** |
| --- | --- | --- | --- | --- | --- | --- | --- | --- |
| Taurocholate | NA | 0 | NA |  | Taurocholate | 0.983 | 44470 | 1.584 |
| Estradiol | NA | 0 | NA |  | Estradiol | 0.949 | 900 | NA |
| L-Aspartate | 0.669 | 118 | NA |  | L-Aspartate | 0.817 | 1170 | NA |
| L-Cystine | 0.826 | 127 | NA |  | L-Cystine | 0.944 | 511 | NA |
| Sphingosine | NA | 0 | NA |  | Sphingosine | 0.986 | 323025 | 2.383 |
| 2-Pyrocatechuic acid | 0.668 | 130 | NA |  | 2-Pyrocatechuic acid | 0.922 | 1204 | NA |
| Glutaconic acid | NA | 0 | NA |  | Glutaconic acid | 0.756 | 178 | NA |
| D-Fructose | NA | 0 | NA |  | D-Fructose | NA | 0 | NA |
| L-Kynurenine | NA | 0 | NA |  | L-Kynurenine | 0.986 | 97598 | 2.581 |
| Lactulose | NA | 0 | NA |  | Lactulose | 0.696 | 228 | NA |
| 3-Hydroxyisovaleric acid | 0.577 | 111 | NA |  | 3-Hydroxyisovaleric acid | 0.959 | 1257 | NA |
| 5alpha-Cholestanol | NA | 0 | NA |  | 5alpha-Cholestanol | NA | 0 | NA |
| Glyceraldehyde | 0.576 | 113 | NA |  | Glyceraldehyde | NA | 0 | NA |
| Coenzyme A | NA | 0 | NA |  | Coenzyme A | 0.577 | 115 | NA |
| D-threo-Isocitric acid | NA | 0 | NA |  | D-threo-Isocitric acid | 0.577 | 106 | NA |
| Menadione | NA | 0 | NA |  | Menadione | NA | 0 | NA |
| 1,5-Anhydrosorbitol | NA | 0 | NA |  | 1,5-Anhydrosorbitol | 0.749 | 311 | NA |
| Hydroxylamine | NA | 0 | NA |  | Hydroxylamine | NA | 0 | NA |
| Quinone | NA | 0 | NA |  | Quinone | 0.965 | 618 | NA |
| dimethyl benzimidazole | NA | 0 | NA |  | dimethyl benzimidazole | 0.985 | 2426045 | 1.763 |

**Table S1. The peak information table of the sample separation using mobile phase A in negative ion mode (left) and positive ion mode (right). It can be retrieved from the results of analysis of mobile phase determination.**

| **Compound** | **MCQ** | **peak_intensity** | **asymmetry_factor** |  | **Compound** | **MCQ** | **peak_intensity** | **asymmetry_factor** |
| --- | --- | --- | --- | --- | --- | --- | --- | --- |
| Taurocholate | 0.639 | 237 | NA |  | Taurocholate | 0.982 | 55834 | 1.869 |
| Estradiol | NA | 0 | NA |  | Estradiol | 0.969 | 1339 | NA |
| L-Aspartate | 0.893 | 350 | NA |  | L-Aspartate | 0.903 | 3808 | NA |
| L-Cystine | 0.964 | 479 | NA |  | L-Cystine | 0.956 | 1026 | NA |
| Sphingosine | NA | 0 | NA |  | Sphingosine | 0.986 | 425412 | 2.498 |
| 2-Pyrocatechuic acid | 0.982 | 448833 | 2.342 |  | 2-Pyrocatechuic acid | NA | 0 | NA |
| Glutaconic acid | 0.923 | 2989 | NA |  | Glutaconic acid | 0.82 | 200 | NA |
| D-Fructose | 0.919 | 7346 | 2.144 |  | D-Fructose | 0.984 | 250087 | 1.813 |
| L-Kynurenine | 0.975 | 6531 | 2.911 |  | L-Kynurenine | 0.986 | 107540 | 2.214 |
| Lactulose | 0.789 | 317 | NA |  | Lactulose | 0.96 | 1801 | NA |
| 3-Hydroxyisovaleric acid | 0.633 | 58731 | NA |  | 3-Hydroxyisovaleric acid | 0.917 | 1483 | NA |
| 5alpha-Cholestanol | NA | 0 | NA |  | 5alpha-Cholestanol | 0.576 | 175 | NA |
| Glyceraldehyde | 0.901 | 8358 | 5.185 |  | Glyceraldehyde | 0.966 | 574 | NA |
| Coenzyme A | 0.932 | 142 | NA |  | Coenzyme A | 0.832 | 283 | NA |
| D-threo-Isocitric acid | NA | 0 | NA |  | D-threo-Isocitric acid | 0.771 | 6126 | NA |
| Menadione | NA | 0 | NA |  | Menadione | 0.983 | 20179 | 2.269 |
| 1,5-Anhydrosorbitol | 0.973 | 1743 | NA |  | 1,5-Anhydrosorbitol | 0.883 | 419 | NA |
| Hydroxylamine | NA | 0 | NA |  | Hydroxylamine | NA | 0 | NA |
| Quinone | NA | 0 | NA |  | Quinone | 0.934 | 722 | NA |
| dimethyl benzimidazole | 0.912 | 4585 | NA |  | dimethyl benzimidazole | 0.984 | 2475391 | 1.33 |

**Table S2. The peak information table of the sample separation using mobile phase B in negative ion mode (left) and positive ion mode (right). It can be retrieved from the results of analysis of mobile phase determination.**

| compound | mcq | rt | std_blk | peak_intensity | rt_rsd | jagedness | asymmetry_factor | FWHM | modality | validation |
| --- | --- | --- | --- | --- | --- | --- | --- | --- | --- | --- |
| Taurocholate | 0.982 | 297.802 | Inf | 14368.963 | 0.102 | 0 | 1.798 | 5.678 | 0 | TRUE |
| Estradiol | 0.953 | 52.112 | 4 | 1028.054 | 1.145 | 0 | 0.618 | 5.072 | 0.192 | FALSE |
| L-Aspartate | 0.927 | 410.124 | 8.703 | 3463.745 | 0.117 | 0 | 5.722 | 5.665 | 0.105 | TRUE |
| L-Cystine | 0.686 | 404.134 | Inf | 74.725 | 86.035 | 0.006 | 0.853 | 2.878 | 0.433 | FALSE |
| Sphingosine | 0.986 | 308.782 | 221.615 | 135709.179 | 0.097 | 0 | 2.336 | 8.609 | 0 | TRUE |
| 2-Pyrocatechuic acid | NA | 0.672 | Inf | 0 | 3.031 | 0 | NA | NA | 0 | FALSE |
| Glutaconic acid | 0.749 | 97.056 | Inf | 95.907 | 45.216 | 0.004 | 1.614 | 2.941 | 0.353 | FALSE |
| D-Fructose | 0.986 | 97.056 | Inf | 233657.128 | 10.319 | 0 | 1.988 | 6.657 | 0 | TRUE |
| L-Kynurenine | 0.985 | 384.664 | Inf | 26104.609 | 0.074 | 0 | 2.384 | 8.627 | 0 | TRUE |
| Lactulose | 0.718 | 255.365 | Inf | 106.073 | 9.949 | 0.006 | 1.112 | 3.202 | 0. 384 | FALSE |
| 3-Hydroxyisovaleric acid | 0.604 | 71.089 | Inf | 74.568 | 15.262 | 0.003 | 1.001 | 2.931 | 0.47 | FALSE |
| 5alpha-Cholestanol | NA | 0.672 | Inf | 0 | 3.031 | 0 | NA | NA | 0 | FALSE |
| Glyceraldehyde | 0.958 | 425.104 | Inf | 683.885 | 0.005 | 0 | 1.618 | 5.482 | 0.159 | FALSE |
| Coenzyme A | 0.743 | 346.231 | Inf | 138.567 | 0.363 | 0 | 0.868 | 2.911 | 0.408 | FALSE |
| D-threo-Isocitric acid | 0.821 | 83.074 | Inf | 140.164 | 2.131 | 0.001 | 2.493 | 7.104 | 0.313 | FALSE |
| Menadione | 0.696 | 60.601 | Inf | 411.76 | 8.328 | 0.001 | 1.213 | 3.134 | 0.324 | FALSE |
| 1,5-Anhydrosorbitol | 0.577 | 391.155 | Inf | 42.939 | 64.364 | 0.002 | 1.001 | 2.975 | 0.506 | FALSE |
| Hydroxylamine | NA | 0.672 | Inf | 0 | 3.031 | 0 | NA | NA | 0 | FALSE |
| Quinone | 0.934 | 425.603 | Inf | 718.453 | 0.068 | 0 | 0.906 | 4.899 | 0.202 | FALSE |
| dimethyl benzimidazole | 0.986 | 267.85 | 434.036 | 1460584.877 | 0.11 | 0 | 2.337 | 11.849 | 0 | TRUE |

**Table S3. The result table of peak evaluation. The eight metrices and retention time of 20 metabolites in the sample are generated**
